# Impact of follicular size categories on oocyte quality at trigger day in young and advanced-age patients undergoing GnRH-ant therapy

**DOI:** 10.3389/fendo.2023.1167395

**Published:** 2023-04-14

**Authors:** Jingwei Yang, Jing Gao, Yuting Wang, Hongya Liu, Xuemei Lian

**Affiliations:** ^1^ Chongqing Key Laboratory of Human Embryo Engineering, Center for Reproductive Medicine, Women and Children’s Hospital of Chongqing Medical University, Chongqing, China; ^2^ Chongqing Clinical Research Center for Reproductive Medicine, Chongqing Health Center for Women and Children, Chongqing, China; ^3^ School of Public Health, Chongqing Medical University, Chongqing, China; ^4^ Department of Academic Affairs, Children's Hospital of Chongqing Medical University, Chongqing, China; ^5^ Department of Medical Records and Statistics, The Second Affiliated Hospital of Chongqing Medical University, Chongqing, China

**Keywords:** follicle size, oocyte quality, advanced age, GnRH-ant cycles, trigger

## Abstract

**Aim:**

To study the effect of follicle sizes of different proportions on oocyte and embryo quality in young and advanced-age patients, and provide evidence for personalized protocol adjustment.

**Methods:**

This was a retrospective real-world data study including a total of 11,462 patients who had started their first *in vitro* fertilization cycle with a gonadotropin-releasing hormone antagonist (GnRH-ant) protocol during 2018–2021. We classified patients into groups according to the size of the dominant proportion of follicles on the human chorionic gonadotropin (hCG) trigger day: Large, Medium, Small, and Equal (containing equivalent proportions of all three size categories). The Cochran–Mantel–Haenszel test by different Anti-Mullerian Hormone (AMH) and antral follicle count (AFC) was used to compare factors such as the metaphase II (MII) oocyte rate, normal fertilization rate, and two pronuclei (2PN) cleavage rate between groups. General linear model (GLM) analysis was performed for inter-group comparison of the oocyte and embryo quality.

**Results:**

In patients aged < 35 years and with AMH ≥ 1.2μg/L, the MII oocyte percentages in the Large and Medium groups were significantly higher than in the Small group (*P* < 0.001). The germinal vesicle (GV) oocyte and unavailable oocyte percentages in the Large and Medium groups were lower than in the Small group (*P* < 0.001). Among patients aged ≥ 35 years with AFC < 5 and AMH ≥ 1.2μg/L, the GV oocyte percentage in the Large group was significantly lower than in the Medium group (2.54% vs. 4.46%, *P* < 0.001). In patients < 35 years, the GLM demonstrated that the Large and Medium groups had positively impacted on the development of MII oocyte and live birth rate(LBR) of first embryo transfer(ET)(β>0, all *P* value < 0.05);and had less likely to develop into unavailable oocyte, degenerated oocyte, GV oocyte and MI oocyte rates relative to the Small group(β<0, all *P* value < 0.05). And among patients ≥ 35 years, the Medium group had positively impacted on the development of MII oocyte and 2PN rates relative to the Small group(β>0, all *P* value < 0.05); and had less likely to develop into MI oocytes relative to the Small group(β<0, all *P* value < 0.05). The GLM indicated that AMH, along with Gn total dose, start dose, and Gn days, had significant impact on oocyte and embryo quality. For young patients, age was not a significant influencing factor, but for advanced-age patients, age influenced the outcomes.

**Conclusion:**

Our analysis suggests that for young patients (< 35 years), triggering when there is a high proportion of large or medium follicles results in better quality oocytes, while for older patients (≥ 35 years), it is better to trigger when the proportion of medium follicles is no less than that of small follicles. Further research is required to confirm these findings.

## 1 Introduction

The main purpose of assisted reproductive technology (ART) treatment is to achieve high-quality oocytes and embryos (1), as oocyte quality is perhaps the important limiting factor in female fertility (2). The controlled ovarian stimulation (COS) process uses exogenous gonadotropins to promote the development of follicles of different sizes so that mature oocytes can be collected, increasing the chance of obtaining a viable embryo (3). To ensure the quality of oocytes, doctors determine the timing of trigger hormone injection based on follicle size, number, and serum hormone levels. However, not all follicles develop in synchrony, and different sizes of follicles can exist at the same time.

COS treatment is based on an assumption that follicular size predicts the quality of oocyte development, fertilization, and cleavage-stage embryo morphology (4, 5). However, there is no consensus in the current literature as to an optimal follicle size for triggering. One widely applied protocol is to start the trigger when several follicles reach a diameter of 17 or 18 mm (6, 7). Other studies indicate triggering at follicle diameters of 12–15 mm (8, 9). Studies have shown that follicles with greater diameter are most likely to yield a mature oocyte that is capable of normal fertilization suited for high-quality embryos, while smaller follicles showed lower fertilization rates (10, 11). Some reports suggest that the proportion of metaphase II (MII) oocytes is significantly lower in small follicles (< 13 mm) than in large follicles (≥ 16 mm) (12, 13). However, other studies found no differences between large follicles (> 18.5 mm) and small follicles (< 14.5 mm) in rates of immature oocyte development, fertilization, and cleavage (5, 14). These inconsistent results regarding the dominant follicle size may be due to differences in patient characteristics. Therefore, the relationship between follicular size and the developmental capacity of oocytes is still controversial.

It is known that many conditions affect oocyte quality, the most important objective condition being the age of the patient and the most important controllable condition being the COS process. In hospitals with a large number of patients, recording the outcome of each follicle and oocyte is a great deal of clinical work. However, when determining optimal follicle size, it is crucial to consider patient characteristics, especially the effect of age. Current studies have shown that trigger criteria for advanced-age patients are still inconclusive (15, 16). Therefore, a clinically applicable indicator is needed to assist physicians in personalizing the trigger.

In an attempt to obtain evidence for personalized protocol adjustment, our study compared oocyte and embryo outcomes of young and advanced-age patients categorized into different groups based on the proportion of follicles of different size on the day of hCG trigger.

## 2 Materials and methods

### 2.1 Study design

This was a retrospective real-world data study linking the information of patients from the electronic medical record system to evaluate women who commenced their first COS with the gonadotropin-releasing hormone antagonist (GnRH-ant) protocol (both *in vitro* fertilization [IVF] and intracytoplasmic sperm injection), including fresh or freeze-all cycles, from January 2018 to June 2021, in Women and Children’s Hospital of Chongqing Medical University.

### 2.2 Participants

All patients included in this study were diagnosed with pelvic environment tubal factor infertility, or male factor infertility, and were undergoing their first COS cycle with the GnRH-ant protocol. Excluded from the data analysis were patients with body mass index(BMI) ≥ 28kg/m^2^, premature ovarian insufficiency(POI) and premature ovarian failure(POF), preimplantation genetic testing cycles, male/female chromosome abnormalities, genetic diseases, polycystic ovarian syndrome, uterine malformation, endometriosis, and serious metabolic and endocrine diseases. A total of 11,462 patients were analyzed, including 11,249 patients who had finished the COS process and undergone oocyte retrieval surgery, and another 213 patients who interrupted the COS treatment or canceled oocyte retrieval for personal reasons.

### 2.3 Data sources

#### 2.3.1 GnRH-ant protocol

COS was performed using the GnRH-ant protocol, as previously described (17). The starting dose was determined by factors including antral follicle count (AFC), age, and body mass index (BMI). A starting dose of 100–149 IU is typically recommended for patients with a very high ovarian response, 150–225 IU for those with normal ovarian response, and 225–300 IU for those with a very poor ovarian response (18). Treatment was initiated on day 2 or 3 of the cycle with a dose of rFSH ranging from 75 to 300 IU. The patients were administered a daily dose of 0.25 mg of GnRH-ant (Orgalutran, Organon, the Netherlands; or Cetrorelix, Merck Serono, Switzerland) if at least one of the following criteria was fulfilled: (i) at least one follicle >14 mm; (ii) serum estrogen level > 600 pg/mL; and (iii) serum luteinizing hormone level > 10 IU/L (18). During the follicular monitoring period, doctors adjusted the GnRH-ant dose (≤ 150 IU) no more than two times according to the follicular growth, commonly on days 4–7.

#### 2.3.2 Follicle measurement

Transvaginal ultrasonography (Hitachi Aloka Medical, Tokyo Japan) was performed by a senior physician every 1–2 days to monitor follicle development during ovarian stimulation and to record the follicle diameter until the hCG trigger day. We used the mean of vertical and horizontal ultrasonic measurements of the follicle (orthogonal diameters) as the size of a follicle. For the first 4 days after GnRH-ant administration, follicles more than 5 mm in diameter were recorded; for days 5–7 after GnRH-ant administration, follicles more than 8 mm in diameter were recorded; and for days 8–9, or day 10 and beyond, follicles more than 10 and 12 mm in diameter were recorded. All ultrasound measurements were checked by another physician.

#### 2.3.3 Groups of follicle size and number on hCG day

For a large sample size, recording the outcome of each follicle and oocyte involves a great amount of clinical work and is hard to achieve. We decided to group the patients by combining follicle size and number, and named each group based on the highest proportion of follicles. Each follicle size was measured and recorded before oocyte aspiration. Follicles were grouped according to size on the hCG day: follicles with diameter <16 mm were designated small, follicles 16–18 mm in diameter were medium, and follicles >18 mm in diameter were large (10). We ensured that the follicles in each patient were monitored for all three groups. Combining follicle size and number, patients were grouped according to the size of the predominant proportion of follicles. There were seven types of groups (Large [predominantly large follicles], Medium [predominantly medium], Small [predominantly small], Large & Small [large and small follicles in equal dominant proportion], Large & Medium [large and medium follicles in equal dominant proportion], Medium & Small [medium and small follicles in equal dominant proportion], and Equal [equivalent proportions of all three size categories]). If there were at least three follicles measuring > 16 mm in diameter, then hCG (Merck Serono, Italy) was administered.

#### 2.3.4 Embryo culture and transfer

After trigger administration, transvaginal oocyte retrieval was performed at 36 h; then, embryo transfer was performed on day 3 following oocyte retrieval. Luteal-phase support with vaginal combined oral progesterone was started immediately after oocyte retrieval. According to national guidelines, most patients received double embryo transfer unless the patient had only one available embryo (19). Surplus available embryos or embryos of freeze-all cycles(owing to ovarian hyperstimulation syndrome, thin endometrium, abnormal blood biochemical index, or personal reasons of patients) were frozen for later transfer.

### 2.4 Outcome measures

The outcome indicators were related to oocyte and embryo quality, including percentage rates for MII oocyte, normal fertilization, two pronuclei (2PN), available embryo, degenerated (atretic) oocyte, germinal vesicle (GV) oocyte, meiosis I (MI) oocyte, abnormal oocyte, and unavailable oocyte.

Rate calculations were as follows: MII oocyte rate = number of MII oocytes/number of retrieval oocyte; normal fertilization rate = number of normal fertilized oocytes/number of MII oocytes; 2PN rate = number of normal cleavage embryos/number of normal fertilized oocytes; available embryo rate = number of available embryos/number of normal cleavage embryos. The denominator of degenerated (atretic) oocyte rate, GV oocyte rate, MI oocyte rate, and abnormal oocyte rate is the number of retrieval oocytes, and the sum of these rates is the unavailable oocyte rate. To calculate the secondary outcome, we used the live birth rate (LBR) of first embryo transfer (ET) (defined as the first live baby born at ≥ 28 weeks of gestation resulting from the first ART cycle, including fresh or first embryo transfer of freeze-all cycles) divided by the number of patients who had started their first ART cycle during the study period.

### 2.5 Statistical methods

Data that showed normal distribution are presented as mean (standard deviation) and the median is here considered descriptive for data of abnormal distribution, as appropriate. Patient age was categorized into two groups: <35 and ≥35 years (20). The AFC was categorized into <5 and ≥5, and the AMH was categorized into < 1.2 ng/mL and ≥1.2 ng/mL (21). The Cochran–Mantel–Haenszel test and Kruskal–Wallis test by different AMH and AFC were used to compare factors such as the MII oocyte rate, normal fertilization rate, and 2PN cleavage rate between groups. We performed general liner model (GLM) analysis for inter-group comparison of the oocyte and embryo outcomes. *P*-values <0.05 were considered statistically significant. All analyses were conducted using Stata version 15.1 (StataCorp LLC, College Station, TX, USA) and RStudio Team 2015 (RStudio, Inc., Boston, MA, USA).

## 3 Results

### 3.1 Participants

**Figure 1:**
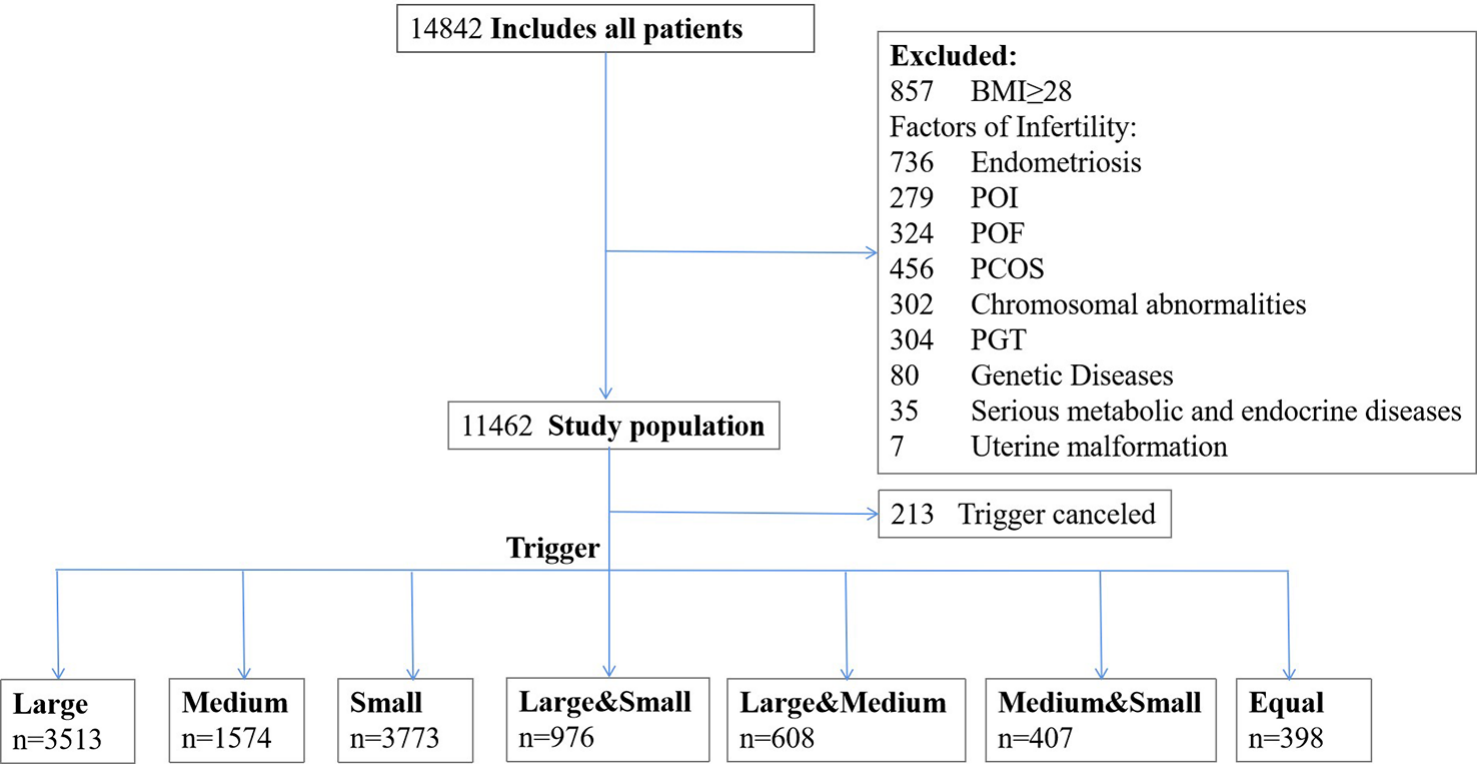
Flow chart.

According to the inclusion and exclusion criteria, a total of 11462 patients were identified as eligible for participation in this analysis (**Figure 1**). After starting COS, 1.86% (213/11462) of patients canceled trigger. **Table 1** summarizes the epidemiological, clinical, and biological characteristics of the study population. The mean age of participants was 33.20 ± 5.09 years, with a range from 20 to 48 years. A mean of 9.70 ± 6.99 oocytes and 3.43 ± 2.81 viable embryos were achieved per patient. Patients were grouped according to the size of the dominant proportion of follicles on the hCG day: Large (30.72%), Medium (13.78%), Small (34.62%), Large & Medium (8.53%), Large & Small (8.54%), Medium & Small (3.55%), and Equal (3.48%).

**Table 1:** Characteristics of patients receiving IVF treatment.

	Large(L)	Medium(M)	Small(S)	Large & Small	Large & Medium	Medium & Small	Equal
N	3513	1574	3773	976	608	407	398
Age(year)	33.59 ± 5.21^a^	32.18 ± 5^b^	32.86 ± 5.02^c^	34.49 ± 4.97^a^	33.64 ± 4.91	32.23 ± 4.75	34.21 ± 4.8^a^
Infertility duration	5.56 ± 4.18^b^	5.47 ± 4.1^b^	5.53 ± 4.16^b^	6.07 ± 4.54^a^	5.68 ± 4.07	5.47 ± 3.95	5.91 ± 4.25
AFC	7.53 ± 5.04^a^	9.6 ± 5.68^c^	8.11 ± 5.36^d^	6.01 ± 4.08^b^	7.25 ± 5.03^a^	8.65 ± 5.29^d^	5.88 ± 4.22^e^
BMI(kg/m^2^)	22.4 ± 2.26	22.52 ± 2.4	22.49 ± 2.32	22.41 ± 2.35	22.29 ± 2.31	22.43 ± 2.43	22.49 ± 2.24
AMH(μg/L)	3.2 ± 3.53^a^	4.52 ± 4.04^b^	3.66 ± 3.65^c^	2.21 ± 2.72^a^	3.1 ± 3.54^a^	3.77 ± 3.61^c^	2.08 ± 2.65^d^
Base FSH(U/L)	6.21 ± 2.63	5.71 ± 2.01	7.16 ± 51.84	6.62 ± 2.3	7.82 ± 31.14	6.15 ± 2.49	6.81 ± 2.43
GN start dose	240.8 ± 65.73^a^	217.85 ± 67.31^b^	233.94 ± 66.43^c^	262.05 ± 56.4^a^	247.41 ± 64.41^a^	230.25 ± 66.71^c^	263.54 ± 54.44^a^
GN dose	2222.2 ± 747.1^a^	1965.8 ± 721.13^b^	2087.99 ± 699.54^c^	2294.88 ± 661.38^a^	2223.97 ± 711.16^a^	2055.54 ± 748.8^c^	2270.58 ± 682.86^a^
GN day	9.04 ± 1.52^a^	8.72 ± 1.41^b^	8.6 ± 1.49^b^	8.61 ± 1.53^b^	8.84 ± 1.46^b^	8.61 ± 1.48^b^	8.48 ± 1.64^b^
Trigger canceled	8(0.23%)	5(0.32%)	195(5.17%)	3(0.31%)	1(0.16%)	0	1(0.25%)
rate of no available oocyte	52(1.50%)^a^	5(0.32%)^b^	22(0.59%)^a^	19(1.99%)	6(1.00%)	2(0.49%)	7(1.79%)
Retrieved oocyte	9.26 ± 6.67^a^	12.95 ± 8.18^c^	9.92 ± 6.82^d^	6.76 ± 5.04^b^	8.73 ± 6.8^a^	10.65 ± 6.86^a^	6.46 ± 5.08^b^
Viable embryo	3.31 ± 2.59^a^	4.57 ± 3.5^b^	3.41 ± 2.76^a^	2.55 ± 2.14^a^	3.2 ± 2.58^a^	3.64 ± 2.99^a^	2.56 ± 2.12^a^

*Superscript letters indicate statistical significance of mean values.

*a vs b vs c vs d, P value <0.05, respectively; a vs a, b vs b, c vs c, same letter means no difference.

After the trigger, the rate of no available oocytes was different for each group: Large (1.50%), Medium (0.32%), Small (0.59%), Large & Medium (1.00%), Large & Small (1.99%), Medium & Small (0.49%), and Equal (1.79%). The rate of no available oocytes in the Medium group was lower than those in the Large and Small groups (0.32% vs. 1.50% and 0.59% respectively; *P* < 0.05). Patients with good conditions showed in the Medium group and there were significant differences in characteristics between groups (**Table 1**).

### 3.2 Details regarding embryology of different groups

Considering their characteristics, we stratified patients into subgroups by age, AMH, and AFC. After stratification, the differences among patients at baseline were almost balanced among groups. In patients younger than 35, the GV oocyte, MI oocyte, MII oocyte, and unavailable oocyte percentages were significantly different among the subgroups.

Among patients aged < 35 years with AFC < 5 and AMH ≥ 1.2, the MII oocyte percentage in the Large group was significantly higher than in the Small group (86.97% vs. 82.04%, *P* < 0.001); the GV oocyte percentage in the Large group was lower than in the Small group (2.93% vs. 5.97%, *P* < 0.001); and the unavailable oocyte percentage in the Large group was lower than in the Small group (13.03% vs. 17.96%, *P* < 0.001).

In patients aged < 35 years with AFC ≥ 5 and AMH ≥ 1.2, the MII oocyte percentages in the Large, Large & Medium, and Medium groups were significantly higher than in the Small group (86.21%, 87.55%, and 87.40% vs. 83.69%, all *P* < 0.001); the GV oocyte percentage in the Large group was lower than in the Medium & Small and Small groups (2.45% vs. 4.09% and 4.30%, all *P* < 0.001); the unavailable oocyte percentages in the Large and Medium groups were lower than in the Small group (13.79% and 12.60% vs. 16.43%, all *P* < 0.001); and the LBR of First ET rates in the Large and Medium groups were higher than in the Small group (59.43% and 56.41% vs. 50.92%, all *P* < 0.01) shown in **Table 2**.

**Table 2:** Stratified analysis of oocyte and embryology quality in groups of follicles size after grouping by AFC and AMH in young patients.

Age<35	Large	Medium	Small	Large & Small	Large & Medium	Medium & Small	Equal
**group 1(AFC<5, AMH<1.2)**	275	72	259	116	71	29	58
MII(%)	1072(88.67%)	315(86.07%)	1133(86.49%)	401(88.13%)	231(87.83%)	122(73.49%)	209(84.96%)
Fertilization(%)	765(71.36%)	216(68.57%)	824(72.73%)	299(74.56%)	182(78.79%)	75(61.48%)	157(75.12%)
2PN(%)	751(98.17%)	213(98.61%)	797(96.72%)	293(97.99%)	181(99.45%)	68(90.67%)	152(96.82%)
Avaible embryo(%)	512(68.18%)	147(69.01%)	537(67.38%)	200(68.26%)	133(73.48%)	49(72.06%)	111(73.03%)
Degenerated oocyte(%)	7(0.58%)	6(1.64%)	17(1.3%)	5(1.1%)	3(1.14%)	1(0.6%)	3(1.22%)
GV oocyte(%)	15(1.24%)^a^	12(3.28%)	30(2.29%)	4(0.88%)	8(3.04%)	12(7.23%)^b^	9(3.66%)
MI oocyte(%)	50(4.14%)	13(3.55%)	74(5.65%)	26(5.71%)	14(5.32%)	14(8.43%)	17(6.91%)
Abnormal oocyte(%)	65(5.38%)	20(5.46%)	56(4.27%)	19(4.18%)	7(2.66%)	17(10.24%)	8(3.25%)
Unavailable oocyte(%)	137(11.33%)	51(13.93%)	177(13.51%)	54(11.87%)	32(12.17%)	44(26.51%)	37(15.04%)
LBR of First ET(%)	100(44.64%)	23(38.98%)	79(35.59%)	33(36.67%)	19(31.15%)	4(18.18%)	18(36.73%)
**group 2(AFC≥5, AMH<1.2)**	250	91	243	70	51	29	35
MII(%)	1451(87.62%)	614(85.16%)	1530(85.67%)	363(83.64%)	244(89.05%)	179(86.06%)	194(85.09%)
Fertilization(%)	1056(72.78%)	473(77.04%)	1128(73.73%)	261(71.9%)	188(77.05%)	131(73.18%)	148(76.29%)
2PN(%)	1025(97.06%)	466(98.52%)	1102(97.7%)	255(97.7%)	182(96.81%)	126(96.18%)	145(97.97%)
Avaible embryo(%)	633(61.76%)	284(60.94%)	685(62.16%)	164(64.31%)	113(62.09%)	73(57.94%)	96(66.21%)
Degenerated oocyte(%)	17(1.03%)	8(1.11%)	21(1.18%)	2(0.46%)	1(0.36%)	1(0.48%)	2(0.88%)
GV oocyte(%)	16(0.97%)^a^	25(3.47%)^b^	48(2.69%)	15(3.46%)	5(1.82%)	4(1.92%)	8(3.51%)
MI oocyte(%)	75(4.53%)	45(6.24%)	98(5.49%)	28(6.45%)	12(4.38%)	14(6.73%)	10(4.39%)
Abnormal oocyte(%)	98(5.92%)	29(4.02%)	89(4.98%)	26(5.99%)	12(4.38%)	10(4.81%)	14(6.14%)
Unavailable oocyte(%)	205(12.38%)	107(14.84%)	256(14.33%)	71(16.36%)	30(10.95%)	29(13.94%)	34(14.91%)
LBR of First ET(%)	108(49.54%)	39(48.75%)	95(44.6%)	31(51.67%)	23(53.49%)	11(44%)	17(56.67%)
**group 3(AFC<5, AMH≥1.2)**	163	93	285	60	30	21	24
MII(%)	1188(86.97%)^a^	820(86.96%)	1991(82.04%)^b^	322(82.56%)	198(90%)	153(94.44%)	95(78.51%)
Fertilization(%)	928(78.11%)	594(72.44%)	1524(76.54%)	239(74.22%)	158(79.8%)	126(82.35%)	71(74.74%)
2PN(%)	908(97.84%)	585(98.48%)	1489(97.7%)	230(96.23%)	154(97.47%)	121(96.03%)	70(98.59%)
Avaible embryo(%)	505(55.62%)	344(58.8%)	884(59.37%)	152(66.09%)	96(62.34%)	80(66.12%)	52(74.29%)
Degenerated oocyte(%)	10(0.73%)	9(0.95%)	21(0.87%)	7(1.79%)	/	/	2(1.65%)
GV oocyte(%)	40(2.93%)^a^	26(2.76%)	145(5.97%)^b^	9(2.31%)	7(3.18%)	1(0.62%)	9(7.44%)
MI oocyte(%)	94(6.88%)	65(6.89%)	179(7.38%)	26(6.67%)	12(5.45%)	4(2.47%)	8(6.61%)
Abnormal oocyte(%)	34(2.49%)	23(2.44%)	91(3.75%)	26(6.67%)	3(1.36%)	4(2.47%)	7(5.79%)
Unavailable oocyte(%)	178(13.03%)^a^	123(13.04%)	436(17.96%)^b^	68(17.44%)	22(10%)	9(5.56%)	26(21.49%)
LBR of First ET(%)	58(42.96%)	36(45.00%)	81(33.47%)	20(40.82%)	10(41.67%)	6(31.58%)	5(26.32%)
**group 4(AFC≥5, AMH≥1.2)**	1356	866	1628	261	204	203	95
MII(%)	16035(86.21%)^a^	12546(87.4%)^a^	18423(83.57%)^b^	2504(84.97%)	2553(87.55%)^a^	2335(83.69%)	903(85.03%)
Fertilization(%)	11900(74.21%)	9416(75.05%)	13631(73.99%)	1854(74.04%)	1863(72.97%)	1776(76.06%)	687(76.08%)
2PN(%)	11625(97.69%)	9236(98.09%)	13306(97.62%)	1809(97.57%)	1829(98.17%)	1736(97.75%)	667(97.09%)
Avaible embryo(%)	6297(54.17%)	5012(54.27%)	7241(54.42%)	1028(56.83%)	973(53.2%)	937(53.97%)	381(57.12%)
Degenerated oocyte(%)	195(1.05%)	122(0.85%)	216(0.98%)	23(0.78%)	18(0.62%)	27(0.97%)	8(0.75%)
GV oocyte(%)	456(2.45%)^a^	378(2.63%)	901(4.09%)^b^	102(3.46%)	72(2.47%)	120(4.3%)^b^	45(4.24%)
MI oocyte(%)	1011(5.44%)	731(5.09%)	1502(6.81%)	179(6.07%)	142(4.87%)	184(6.59%)	67(6.31%)
Abnormal oocyte(%)	902(4.85%)	577(4.02%)	1004(4.55%)	139(4.72%)	131(4.49%)	124(4.44%)	39(3.67%)
Unavailable oocyte(%)	2564(13.79%)^a^	1808(12.6%)^a^	3623(16.43%)^b^	443(15.03%)	363(12.45%)	455(16.31%)	159(14.97%)
LBR of First ET(%)	703(59.43%)^a^	418(56.41%)^a^	718(50.92%)^b^	122(52.59%)	94(55.95%)	111(61.33%)	45(54.88%)

*Superscript lettels indicate statistical significance of mean values.

Group1: GV oocyte(%): Large vs Medium&Small: 1.24% vs 7.23%, OR=0.16(95%CI:0.07-0.35), P value<0.001.

Group2: GV oocyte(%): Large vs Medium: 0.97% vs 3.47%, OR=0.27(95%CI:0.14-0.51), P value<0.001.

Group3: MII oocyte(%): Large vs Small: 86.97% vs 82.04%, OR=1.46(95%CI:1.21-1.77), P value<0.001.

GV oocyte(%): Large vs Small: 2.93% vs 5.97%, OR=0.48(95%CI:0.33-0.68), P value<0.001.

Unavailable oocyte(%): Large vs Small: 13.03% vs 17.96%, OR=0.68(95%CI:0.57-0.83), P value<0.001.

Group4: MII oocyte(%): Large vs Small: 86.21% vs 83.57%, OR=1.22(95%CI:1.09-1.36), P value<0.001; Large&Medium vs Small: 87.55 vs 83.57%, OR=1.37(95%CI:1.81-1.59), P value<0.001; Medium vs Small: 87.40% vs 83.57%, OR=1.35(95%CI:1.21-1.51), P value<0.001.

GV oocyte(%): Large vs Small: 2.45% vs 4.09%,OR=0.60(95%CI:0.53-0.66), P value<0.001; Large vs Medium&Small: 2.45% vs 4.30%, OR=0.56(95%CI:0.46-0.69), P value<0.001.

MI oocyte(%): Large vs Small: 5.44% vs 6.81%,OR=0.79(95%CI:0.72-0.85), P value<0.001; Medium vs Small: 5.09% vs 6.81%, OR=0.73(95%CI:0.67-0.80), P value<0.001; Large&Medium vs Small: 4.87% vs 6.81%,OR=0.70(95%CI:0.59-0.84), P value<0.001.

Uavailable oocyte(%): Large vs Small: 13.79% vs 16.43%,OR=0.81(95%CI:0.77-0.86), P value<0.001; Medium vs Small: 12.60% vs 16.43%, OR=0.73(95%CI:0.69-0.78), P value<0.001.

LBR of First ET(%): Large vs Small: 59.43% vs 50.92%,OR=1.21(95%CI:1.11-1.32), P value<0.001; Medium vs Small: 56.41% vs 50.92%, OR=1.13(95%CI:1.02-1.24), P value<0.01.

Among patients aged 35 years and older with AFC < 5 and AMH ≥ 1.2, the GV oocyte percentage in the Large group was significantly lower than in the Medium group (2.54% vs. 4.46%, *P* < 0.001) shown in **Table 3**.

**Table 3:** Stratified analysis of oocyte and embryology quality in groups of follicles size after grouping by AFC and AMH in advanced-age patients.

Age≥35	Large	Medium	Small	Large & Small	Large & Medium	Medium & Small	Equal
**group 1(AFC<5, AMH<1.2)**	519	98	368	212	90	28	81
MII(%)	1928(90.01%)	366(86.94%)	1408(85.91%)	653(88.24%)	307(90.83%)	102(88.7%)	213(86.94%)
Fertilization(%)	1428(74.07%)	286(78.14%)	1039(73.79%)	487(74.58%)	212(69.06%)	69(67.65%)	159(74.65%)
2PN(%)	1403(98.25%)	277(96.85%)	1002(96.44%)	479(98.36%)	205(96.7%)	69(100%)	152(95.6%)
Avaible embryo(%)	934(66.57%)	187(67.51%)	677(67.56%)	340(70.98%)	142(69.27%)	42(60.87%)	119(78.29%)
Degenerated oocyte(%)	18(0.84%)	4(0.95%)	18(1.1%)	5(0.68%)	6(1.78%)	/	5(2.04%)
GV oocyte(%)	25(1.17%)	6(1.43%)	48(2.93%)	16(2.16%)	4(1.18%)	2(1.74%)	6(2.45%)
MI oocyte(%)	79(3.69%)	17(4.04%)	89(5.43%)	39(5.27%)	8(2.37%)	2(1.74%)	9(3.67%)
Abnormal oocyte(%)	96(4.48%)	28(6.65%)	77(4.7%)	27(3.65%)	13(3.85%)	9(7.83%)	12(4.9%)
Unavailable oocyte(%)	214(9.99%)	55(13.06%)	231(14.09%)	87(11.76%)	31(9.17%)	13(11.3%)	32(13.06%)
LBR of First ET(%)	88(22.56%)	13(16.05%)	70(23.81%)	26(17.57%)	13(18.06%)	4(18.18%)	16(25%)
**group 2(AFC≥5, AMH<1.2)**	287	73	223	82	42	21	31
MII(%)	1538(88.7%)	437(92.98%)	1278(88.87%)	361(85.14%)	240(86.02%)	148(90.24%)	153(89.47%)
Fertilization(%)	1101(71.59%)	308(70.48%)	934(73.08%)	243(67.31%)	175(72.92%)	110(74.32%)	116(75.82%)
2PN(%)	1075(97.64%)	305(99.03%)	916(98.07%)	229(94.24%)	173(98.86%)	106(96.36%)	115(99.14%)
Avaible embryo(%)	681(63.35%)	195(63.93%)	553(60.37%)	164(71.62%)	107(61.85%)	63(59.43%)	78(67.83%)
Degenerated oocyte(%)	20(1.15%)	2(0.43%)	11(0.76%)	7(1.65%)	2(0.72%)	1(0.61%)	2(1.17%)
GV oocyte(%)	29(1.67%)	11(2.34%)	26(1.81%)	4(0.94%)	5(1.79%)	4(2.44%)	2(1.17%)
MI oocyte(%)	71(4.09%)	10(2.13%)	66(4.59%)	29(6.84%)	15(5.38%)	8(4.88%)	7(4.09%)
Abnormal oocyte(%)	78(4.5%)	20(4.26%)	58(4.03%)	23(5.42%)	17(6.09%)	3(1.83%)	7(4.09%)
Unavailable oocyte(%)	196(11.3%)	33(7.02%)	160(11.13%)	63(14.86%)	39(13.98%)	16(9.76%)	18(10.53%)
LBR of First ET(%)	67(27.92%)	15(26.79%)	64(32.32%)	14(20.59%)	11(30.56%)	5(31.25%)	5(20.83%)
**group 3(AFC<5, AMH≥1.2)**	202	63	269	70	38	23	37
MII(%)	993(86.95%)	457(84.94%)	1400(84.8%)	307(81%)	220(92.83%)	123(83.11%)	135(83.85%)
Fertilization(%)	731(73.62%)	342(74.84%)	1058(75.57%)	225(73.29%)	157(71.36%)	90(73.17%)	90(66.67%)
2PN(%)	715(97.81%)	331(96.78%)	1022(96.6%)	220(97.78%)	149(94.9%)	89(98.89%)	86(95.56%)
Avaible embryo(%)	465(65.03%)	187(56.5%)	618(60.47%)	144(65.45%)	92(61.74%)	51(57.3%)	67(77.91%)
Degenerated oocyte(%)	11(0.96%)	9(1.67%)	20(1.21%)	4(1.06%)	/	3(2.03%)	4(2.48%)
GV oocyte(%)	29(2.54%)^a^	24(4.46%)^b^	69(4.18%)	21(5.54%)	3(1.27%)	4(2.7%)	/
MI oocyte(%)	44(3.85%)	26(4.83%)	100(6.06%)	16(4.22%)	10(4.22%)	4(2.7%)	8(4.97%)
Abnormal oocyte(%)	65(5.69%)	22(4.09%)	62(3.76%)	31(8.18%)	4(1.69%)	14(9.46%)	14(8.7%)
Unavailable oocyte(%)	149(13.05%)	81(15.06%)	251(15.2%)	72(19%)	17(7.17%)	25(16.89%)	26(16.15%)
LBR of First ET(%)	31(18.79%)	13(26%)	46(20.72%)	9(15.52%)	12(36.36%)	2(9.52%)	5(20%)
**group 4(AFC≥5, AMH≥1.2)**	461	218	498	105	82	53	37
MII(%)	4073(86.73%)	2205(85.76%)	4291(84.19%)	691(82.95%)	703(89.9%)	515(88.49%)	284(85.8%)
Fertilization(%)	3007(73.83%)	1619(73.42%)	3128(72.9%)	507(73.37%)	506(71.98%)	365(70.87%)	199(70.07%)
2PN(%)	2959(98.4%)	1568(96.85%)	3071(98.18%)	488(96.25%)	498(98.42%)	355(97.26%)	193(96.98%)
Avaible embryo(%)	1583(53.5%)	819(52.23%)	1672(54.44%)	286(58.61%)	285(57.23%)	182(51.27%)	108(55.96%)
Degenerated oocyte(%)	53(1.13%)	19(0.74%)	45(0.88%)	11(1.32%)	2(0.26%)	3(0.52%)	2(0.6%)
GV oocyte(%)	114(2.43%)	99(3.85%)	213(4.18%)	44(5.28%)	14(1.79%)	15(2.58%)	11(3.32%)
MI oocyte(%)	223(4.75%)	133(5.17%)	331(6.49%)	48(5.76%)	41(5.24%)	25(4.3%)	22(6.65%)
Abnormal oocyte(%)	233(4.96%)	115(4.47%)	217(4.26%)	39(4.68%)	22(2.81%)	24(4.12%)	12(3.63%)
Unavailable oocyte(%)	623(13.27%)	366(14.24%)	806(15.81%)	142(17.05%)	79(10.1%)	67(11.51%)	47(14.2%)
LBR of First ET(%)	123(30.98%)	74(38.74%)	141(32.94%)	22(23.66%)	30(40.00%)	20(41.67%)	14(42.42%)

*Superscript lettels indicate statistical significance of mean values.

Group 3: GV oocyte(%): Large vs Medium: 2.54% vs 4.46%, OR=0.18(95%CI:0.10-0.33), P value<0.001.

### 3.3 Multivariate analysis of the generalized linear model

**Tables 4**, **5** presents the results of GLM analysis, adjusted for the effects of follicle size groups and covariates (age, AMH, AFC, infertility duration, GnRH-ant total dose, GnRH-ant start dose, GnRH-ant days, retrieval oocytes) on oocyte and embryo quality and IVF outcomes in the young and advanced-age categories.

**Table 4:** GLM analysis of oocyte and embryology quality between groups in young patients.

	Small(S)	Large(L)	Medium(M)	Large & Small	Large & Medium	Medium & Small	Equal
Unavailable oocyte(%)	Ref	-0.022^*^	-0.035^*^	-0.010	-0.044^*^	-0.013	-0.007
Degenerated oocyte(%)	Ref	-0.001	-0.002	/	-0.006^*^	-0.002	0.001
GV oocyte(%)	Ref	-0.010^*^	-0.010^*^	-0.004	-0.007	0.001	0.002
MI oocyte(%)	Ref	-0.013^*^	-0.017^*^	-0.009	-0.021^*^	-0.007	-0.004
abnormal oocyte(%)	Ref	0.002	-0.006	0.004	-0.010	-0.004	-0.006
MII oocyte(%)	Ref	0.022^*^	0.035^*^	0.010	0.044^*^	0.013	0.007
Fertilization(%)	Ref	0.004	-0.001	-0.002	0.001	0.006	0.024
2PN(%)	Ref	0.002	0.009^*^	-0.002	0.005	-0.011	/
avaible embryo(%)	Ref	-0.002	0.009	0.015	0.019	-0.003	0.040
LBR of First ET	Ref	0.257^*^	0.167^*^	0.068	0.135	0.259	0.172

*Superscript symbols indicate statistical significance of mean values, the Small group was included in the GLM as a reference group in the model.

Unavailable oocyte(%): Large: β=-0.022(95%CI:-0.033~-0.011); Medium: β=-0.035(95%CI:-0.049~-0.022);Large & Medium: β=-0.044(95%CI:-0.064~-0.023); all P value<0.001.

Degenerated oocyte(%):Large & Medium: β=-0.006(95%CI:-0.011~-0.001); P =0.016.

GV oocyte(%): Large: β =-0.010(95%CI:-0.015~-0.005); Medium: β=-0.010(95%CI:-0.016~-0.003); all P value<0.005.

MI oocyte(%): Large: β=-0.013(95%CI:-0.020~-0.006); Large & Medium: β=-0.021(95%CI:-0.033~-0.008); Medium: β=-0.017(95%CI:-0.026~-0.009); all P value<0.005.

MII oocyte(%): Large: β=0.022(95%CI:0.011~0.033); Large & Medium: β=0.044(95%CI:0.023~0.064);Medium: β=0.035(95%CI:0.022~0.049); all P value<0.001.

2PN(%): Medium: β=0.009(95%CI:0.001~0.016); P value=0.022.

LBR of First ET(%): Large: β =0.257, OR=1.293(95%CI:1.125~1.487); Large & Medium: β=0.167, OR=1.182(95%CI:1.001~1.399); all P value<0.05.

**Table 5:** GLM analysis of oocyte and embryology quality between groups in advanced-age patients.

	Small(S)	Large(L)	Medium(M)	Large & Small	Large & Medium	Medium & Small	Equal
Unavailable oocyte(%)	Ref	-0.009	-0.01	0.012	-0.021	-0.012	0.008
Degenerated oocyte(%)	Ref	0.002	-0.001	0.005	-0.003	/	0.007
GV oocyte(%)	Ref	-0.008^*^	-0.002	-0.003	-0.008	-0.002	-0.010
MI oocyte(%)	Ref	-0.010^*^	-0.013^*^	0.001	-0.008	-0.028^*^	-0.012
Abnormal oocyte(%)	Ref	0.001	/	0.006	-0.006	0.005	0.015
MII oocyte(%)	Ref	-0.003	0.034*	-0.018	0.006	0.064*	-0.006
Fertilization(%)	Ref	-0.010	0.030	-0.020	-0.013	0.022	-0.011
2PN(%)	Ref	-0.008	0.022	-0.014	-0.008	0.060*	0.008
Avaible embryo(%)	Ref	-0.013	0.031	-0.004	0.019	0.010	0.018
LBR of First ET	Ref	-0.085	-0.014	-0.297	0.082	-0.213	0.114

*Superscript symbols indicate statistical significance of mean values, the Small group was included in the GLM as a reference group in the model.

GV oocyte(%): Large: β =-0.008(95%CI:-0.014~-0.002); P value=0.007.

MI oocyte(%): Large: β=-0.010(95%CI:-0.019~-0.002); Medium: β=-0.013(95%CI:-0.025~-0.001); Medium & Small: β=-0.028(95%CI:-0.049~-0.007); all P value<0.05.

MII oocyte(%): Medium: β=0.034(95%CI:0.003~0.066); Medium & Small: β=0.064(95%CI:0.010~0.118); all P value<0.05.

2PN(%): Medium & Small: β=0.060(95%CI:0.007~0.114); P value=0.026.

Considering the results of CMH analysis, differences between groups existed mainly between the Small group and other groups, so the Small group was included as a reference group in the GLM.

#### 3.3.1 Factors influencing outcomes of IVF among patients aged < 35 years

Among patients younger than 35 years, as shown in **Table 4**, the Large, Medium, and Large & Medium groups were positively impact on the development of MII oocyte (Large: β=0.022; Large & Medium: β=0.044; Medium: β=0.035; all *P* < 0.001), 2PN (Medium: β=0.009; *P* = 0.022) and LBR of First ET(Large: β = 0.257; Large & Medium: β = 0.167; all *P* < 0.05) relative to the Small group.

On the contrary, the Large, Medium, and Large & Medium groups had less likely to develop into unavailable oocyte (Large: β=−0.022; Medium: β=−0.035; Large & Medium: β=−0.044; all *P* < 0.001), degenerated oocyte(Large & Medium: β=−0.006; *P* =0.016), GV oocyte (Large: β=−0.010; Medium: β=−0.010; all *P* < 0.005) and MI oocyte (Large: β=−0.013; Large & Medium: β=−0.021; Medium: β=−0.017; all *P* < 0.005) relative to the Small group. The GLM also demonstrated that the higher the AMH, the better the IVF outcome. Additionally, the Gn dose, start dose, and Gn days of COS process impacted the oocyte quality and IVF outcome (**Table 6**).

**Table 6:** Factors influencing oocyte and embryology quality between groups.

Coefficients (95%CI)	<35 years					≥35 years				
	Age	AMH	Gn dose	Gn start	Gn days	Age	AMH	Gn dose	Gn start	Gn days
Unavailable oocyte(%)	/	-0.002 (-0.003~-0.001)	/	/	-0.031 (-0.039~-0.022)	0.020 (0.018~ 0.022)	/	0.002 (0.001~0.003)	-0.001 (-0.002~-0.001)	-0.042 (-0.061~-0.022)
Degenerated oocyte(%)	/	/	/	/	/	0.002 (0.001~ 0.003)	/	/	/	/
GV oocyte(%)	/	-0.002 (-0.003~-0.001)	0.003 (0.001~0.005)	/	-0.015 (-0.019~-0.011)	0.006 (0.003~ 0.010)	-0.002 (-0.003~-0.001)	/	/	-0.012 (-0.022~-0.003)
MI oocyte(%)	/	/	0.003 (0.001~0.005)	/	-0.014 (-0.019~-0.008)	/	/	0.009 (0.003~0.015)	/	-0.035 (-0.048~-0.022)
Abnormal oocyte(%)	/	/	/	/	/	0.008 (0.006~ 0.009)	/	0.004 (0.001~0.007)	0.002 (0.001~0.003)	/
MII oocyte(%)	/	0.002 (0.001~0.003)	-0.011 (-0.024~-0.002)	/	0.031 (0.022~0.039)	-0.128 (-0.131~ -0.124)	0.007 (0.002~0.014)	0.001 (0.0003~0.0013)	0.002 (0.001~0.003)	0.052 (0.017~0.086)
Fertilization(%)	/	/	/	/	/	-0.110 (-0.113~ -0.106)	/	/	/	/
2PN(%)	/	/	/	/	/	-0.144 (-0.148~ -0.141)	/	/	/	/
Avaible embryo(%)	/	/	/	/	/	-0.099 (-0.103~ -0.096)	/	/	/	/
LBR of First ET	/	0.034 (0.019~0.049)	0.002 (0.001~0.003)	-0.004 (-0.007~-0.001)	0.133 (0.020~0.247)	-0.253 (-0.289~-0.217)	0.039 (0.016~0.061)	/	/	/

All coefficients(95%CI) shown in the table are statistically significant in GLM.

#### 3.3.2 Factors influencing outcomes of IVF among patients aged ≥ 35 years

Among patients aged 35 years and older, as shown in **Table 5**, the Medium and Medium & Small groups were positively impact on the development of MII oocyte (Medium: β=0.034; Medium & Small: β=0.064; all *P* < 0.05) and 2PN (Medium & Small: β=0.060; *P* value=0.026) relative to the Small group. In contrast, the Large, Medium, and Medium & Small groups had less likely to develop into GV oocyte (Large: β =−0.008; *P* = 0.007) and MI oocyte (Large: β=−0.010; Medium: β=−0.013; Medium & Small: β=−0.028; all *P* < 0.05) relative to the Small group. And the GLM still suggests that age remains the most important influencing factor for patients of advanced age. After 35 years old, elevated age negatively affects oocyte quality and life birth. Also, AMH, Gn dose, Gn start dose and Gn days of COS process impacted the oocyte quality (**Table 6**).

## 4 Discussion

During COS monitoring, doctors administer the trigger for oocyte maturation once the appropriate number and size of follicles are reached ^9^. However, in our study, instead of focusing on just several dominant follicle sizes, we considered the overall follicular development in conjunction with the patient’s age to decide about the trigger time. Our results showed that for patients aged younger than 35, administering the trigger when there was a high proportion of large or medium follicles resulted in higher quality oocytes, while patients 35 and older had better results when the proportion of medium follicles was no less than that of small follicles at trigger time.

A widely used protocol is to trigger final oocyte maturation when 2 or 3 lead follicles reach or exceed 17–18 mm in diameter. However, not all follicles develop in synchrony, and the diameters of the entire set of punctured follicles can range from < 12 mm to >28 mm on retrieval about 36 h after trigger (22). Many studies have previously discussed that administration of hCG at smaller follicle sizes can result in follicular atresia or ovulation of immature oocytes (23, 24). We found a significantly lower rate of available oocytes from small follicles than from large follicles, and the GV and MI rates were significantly higher in the group with predominantly small follicles (<16 mm) than in the group with predominantly large or medium follicles (≥16 mm), which is consistent with previous studies (25, 26). Mohr-Sasson et al. (27) found MII oocytes more commonly in the medium and large follicle groups, indicating that follicles ≥15 mm in diameter have the highest probability of producing mature oocytes. Further, Nogueira et al. (28) found that mature oocytes retrieved from small follicles (<12 mm) generated embryos of lower developmental potential than oocytes derived from larger follicles. A retrospective study demonstrated that the exposure of small follicles (<16 mm) to ovulatory trigger results in a heterogeneous collection of MII-stage and MI-stage oocytes (29). Therefore, the decreased LBR of first ET of oocytes from the Small group in our analysis may be due to a lower proportion of mature oocytes. However, there was no significant difference in the normal fertilization rate between groups; in other words, oocytes from small follicles have the same potential for fertilization as oocytes from large follicles if they are mature (12).

The most interesting finding in our study was the different optimal follicle sizes for trigger in young and advanced-age patients. Previous studies have racked the outcome of each oocyte (12, 22), and reported the effect of follicle size on oocyte and embryo quality (30–32). In our study, instead of focusing on the dominant follicles, we focused on the overall proportion of follicle size and personalized the trigger by combining it with age characteristics. It showed that if triggered when there was a higher proportion of large or medium follicles might obtain better quality oocytes for young patients, while in advanced-age patients, it is best to wait until the proportion of medium follicles is not lower than that of small follicles to perform the trigger. Such differences may result from decreased follicular output rate, the follicle–oocyte index, and the ovarian sensitivity index (33–36) in older patients. Therefore, promoting follicle growth is more difficult in advanced-age patients than in young patients.

The GLM analysis indicated that AMH, along with GnRH-ant total dose, start dose, and Gn days, significantly impacted oocyte and embryo quality. However, it is noteworthy that after separating the patients by age group, age was no longer a significant influencing factor in patients younger than 35, but in the group of patients 35 and older, age still influenced the outcomes. The ability of women to produce oocytes of good quality and quantity decreases with age due to decreased ovarian reserve and response to ovarian stimulation. Many studies have used markers such as the decreased level of AMH, estradiol, and AFC to assess the impact of aging on ovarian reserve (37, 38) while other research has found an increase in basal FSH (39). Other studies based on genetic evidence show that advanced age can lead to a progressive decrease in oocyte/embryo competence: women ≥ 35 years old experience a dramatic increase in embryo aneuploidy rate, from 30% up to 90% (40, 41), and women 40 years and older have a higher chance (> 80%) of producing a blastocyst with chromosomal abnormalities (42). Our study suggests that the COS process (GnRH-ant total dose, start dose, and days) affects oocyte and embryo quality. Use of our data to improve treatment protocols may allow patients to obtain more high-quality oocytes. However, the limitations of advanced age present an open dilemma and a continuing challenge for clinicians working in the field of ART.

### 4.1 Strengths and limitations

Our results provide evidence for the optimal proportion of follicle size on the hCG trigger day in young and advanced-age patients. This could assist physicians in making clinical decisions for personalized trigger and protocol adjustment. The limitations of this study include its retrospective nature. Because we analyzed real-world data, the baseline characteristics were unbalanced between groups. Additionally, by the end of our research, many patients had embryos left for transfer. A well-designed, multicenter study is still warranted to further support our results.

## 5 Conclusion

Our results suggest that in young patients (< 35 years) the optimal time to trigger is when there was a high proportion of large or medium follicles, while in advanced-age patients (≥ 35 years) when the proportion of medium follicles is no less than that of small follicles, triggering is likely to result in better-quality oocytes. There is further opportunity for study in determining how to improve the performance of follicle groups by regulating stimulation.

## Data availability statement

The original contributions presented in the study are included in the article/supplementary material. Further inquiries can be directed to the corresponding author.

## Author contributions

The present work was designed by JY and XL. Data extraction and analysis were performed by JY, JG and YW. JY and HL participated in the data collection. JY, JG and XL made revisions to the article. All authors contributed to the article and approved the submitted version.

## References

[1] Colaco S Sakkas D. Paternal factors contributing to embryo quality. J Assist Reprod Genet (2018) 35(11):1953–68. doi: 10.1007/s10815-018-1304-4 PMC6240539 30206748

[2] Gilchrist RB Lane M Thompson JG. Oocyte-secreted factors: regulators of cumulus cell function and oocyte quality. Hum Reprod Update (2008) 14(2):159–77. doi: 10.1093/humupd/dmm040 18175787

[3] Hancock KL Pereira N Christos PJ Petrini AC Hughes J Chung PH. Optimal lead follicle size for human chorionic gonadotropin trigger in clomiphene citrate and intrauterine insemination cycles: an analysis of 1,676 treatment cycles. Fertil Steril (2021) 115(4):984–90. doi: 10.1016/j.fertnstert.2020.10.026 33272641

[4] Wirleitner B Okhowat J Vištejnová L Králíčková M Karlíková M Vanderzwalmen P. Relationship between follicular volume and oocyte competence, blastocyst development and live-birth rate: optimal follicle size for oocyte retrieval. Ultrasound Obstet Gynecol (2018) 51(1):118–25. doi: 10.1002/uog.18955 29134715

[5] Madani T Mohammadi Yeganeh L Ezabadi Z Hasani F Chehrazi M. Comparing the efficacy of urinary and recombinant hCG on oocyte/follicle ratio to trigger ovulation in women undergoing intracytoplasmic sperm injection cycles: a randomized controlled trial. J Assist Reprod Genet (2013) 30(2):239–45. doi: 10.1007/s10815-012-9919-3 PMC3585675 23274511

[6] Fauque P Lehert P Lamotte M Bettahar-Lebugle K Bailly A Diligent C. Clinical success of intrauterine insemination cycles is affected by the sperm preparation time. Fertil Steril (2014) 101(6):1618–23e1-3. doi: 10.1016/j.fertnstert.2014.03.015 24745729

[7] Loumaye E Engrand P Shoham Z Hillier SG Baird DT. Clinical evidence for an LH 'ceiling' effect induced by administration of recombinant human LH during the late follicular phase of stimulated cycles in world health organization type i and type II anovulation. Hum Reprod (2003) 18(2):314–22. doi: 10.1093/humrep/deg066 12571167

[8] Abbara A Vuong LN Ho VNA Clarke SA Jeffers L Comninos AN. Follicle size on day of trigger most likely to yield a mature oocyte. Front Endocrinol (Lausanne) (2018) 9:193. doi: 10.3389/fendo.2018.00193 29743877 PMC5930292

[9] Mehri S Levi Setti PE Greco K Sakkas D Martinez G Patrizio P. Correlation between follicular diameters and flushing versus no flushing on oocyte maturity, fertilization rate and embryo quality. J Assist Reprod Genet (2014) 31(1):73–7. doi: 10.1007/s10815-013-0124-9 PMC3909131 24189964

[10] Rosen MP Shen S Dobson AT Rinaudo PF McCulloch CE Cedars MI. A quantitative assessment of follicle size on oocyte developmental competence. Fertil Steril (2008) 90(3):684–90. doi: 10.1016/j.fertnstert.2007.02.011 PMC4624406 18249377

[11] Revelli A Martiny G Delle Piane L Benedetto C Rinaudo P Tur-Kaspa I. A critical review of bi-dimensional and three-dimensional ultrasound techniques to monitor follicle growth: do they help improving IVF outcome? Reprod Biol Endocrinol (2014) 12:107. doi: 10.1186/1477-7827-12-107 25420733 PMC4255967

[12] Tamura I Kawamoto-Jozaki M Fujimura T Doi-Tanaka Y Takagi H Shirafuta Y. Relationship between follicular size and developmental capacity of oocytes under controlled ovarian hyperstimulation in assisted reproductive technologies. Reprod Med Biol (2021) 20(3):299–304. doi: 10.1002/rmb2.12382 34262397 PMC8254166

[13] Triwitayakorn A Suwajanakorn S Pruksananonda K Sereepapong W Ahnonkitpanit V. Correlation between human follicular diameter and oocyte outcomes in an ICSI program. J Assist Reprod Genet (2003) 20(4):143–7. doi: 10.1023/a:1022977002954 PMC3455636 12762412

[14] Akbariasbagh F Lorzadeh N Azmoodeh A Ghaseminejad A Mohamadpoor J Kazemirad S. Association among diameter and volume of follicles, oocyte maturity, and competence in intracytoplasmic sperm injection cycles. Minerva Ginecol (2015) 67(5):397–403. 26491821

[15] Zhou C Yang X Wang Y Xi J Pan H Wang M. Ovulation triggering with hCG alone, GnRH agonist alone or in combination? a randomized controlled trial in advanced-age women undergoing IVF/ICSI cycles. Hum Reprod (2022) 37(8):1795–805. doi: 10.1093/humrep/deac114 35595223

[16] Rahav Koren R Miller N Moran R Decter D Berkowitz A Haikin Herzberger E. GnRH agonist-triggering ovulation in women with advanced age. Sci Rep (2022) 12(1):16401. doi: 10.1038/s41598-022-20619-4 36180515 PMC9525572

[17] Yang J Zhang X Ding X Wang Y Huang G Ye H. Cumulative live birth rates between GnRH-agonist long and GnRH-antagonist protocol in one ART cycle when all embryos transferred: real-word data of 18,853 women from china. Reprod Biol Endocrinol (2021) 19(1):124. doi: 10.1186/s12958-021-00814-0 34384445 PMC8359059

[18] Li R. Expert consensus on the standardized application of GnRH-ant protocol in assisted reproduction technology. Chin J Reprod Contracep (2022) 42(2):109–16. doi: 10.3760/cma.j.cn101441-20211108-00495

[19] Sun YJ Huang GN Sun HX Fan LQ Shen H Liu P. Chinese expert consensus on numbers of embryos transferred. J Reprod Med (2018) 27:940–5. doi: 10.3969/j.issn.1004-3845.2018.10.003

[20] Ledger W. Preimplantation genetic screening should be used in all *in vitro* fertilisation cycles in women over the age of 35 years: AGAINST: Pre-implantation genetic screening should not be used in all IVF cycles in women over the age of 35 years. BJOG (2019) 126(13):1555. doi: 10.1111/1471-0528.15942 31617297

[21] Chinta P Antonisamy B Mangalaraj AM Kunjummen AT Kamath MS. POSEIDON classification and the proposed treatment options for groups 1 and 2: time to revisit? a retrospective analysis of 1425 ART cycles. Hum Reprod Open (2021) 2021(1):hoaa070. doi: 10.1093/hropen/hoaa070 33614989 PMC7882041

[22] Shapiro BS Rasouli MA Verma K Raman A Garner FC Aguirre M. The effect of ovarian follicle size on oocyte and embryology outcomes. Fertil Steril (2022) 117(6):1170–6. doi: 10.1016/j.fertnstert.2022.02.017 35367061

[23] Kosmas IP Tatsioni A Fatemi HM Kolibianakis EM Tournaye H Devroey P. Human chorionic gonadotropin administration vs. luteinizing monitoring for intrauterine insemination timing, after administration of clomiphene citrate: a meta-analysis. Fertil Steril (2007) 87(3):607–12. doi: 10.1016/j.fertnstert.2006.10.003 17173907

[24] Farhi J Orvieto R Gavish O Homburg R. The association between follicular size on human chorionic gonadotropin day and pregnancy rate in clomiphene citrate treated polycystic ovary syndrome patients. Gynecol Endocrinol (2010) 26(7):546–8. doi: 10.3109/09513591003686312 20218821

[25] Salha O Nugent D Dada T Kaufmann S Levett S Jenner L. The relationship between follicular fluid aspirate volume and oocyte maturity in in-vitro fertilization cycles. Hum Reprod (1998) 13(7):1901–6. doi: 10.1093/humrep/13.7.1901 9740446

[26] Bergh C Broden H Lundin K Hamberger L. Comparison of fertilization, cleavage and pregnancy rates of oocytes from large and small follicles. Hum Reprod (1998) 13(7):1912–5. doi: 10.1093/humrep/13.7.1912 9740448

[27] Mohr-Sasson A Orvieto R Blumenfeld S Axelrod M Mor-Hadar D Grin L. The association between follicle size and oocyte development as a function of final follicular maturation triggering. Reprod BioMedicine Online (2020) 40(6):887–93. doi: 10.1016/j.rbmo.2020.02.005 32389425

[28] Nogueira D Friedler S Schachter M Raziel A Ron-El R Smitz J. Oocyte maturity and preimplantation development in relation to follicle diameter in gonadotropin-releasing hormone agonist or antagonist treatments. Fertil Steril (2006) 85(3):578–83. doi: 10.1016/j.fertnstert.2005.08.033 16500322

[29] Chen L Jiang S Xi Q Li W Lyu Q Kuang Y. Optimal lead follicle size in letrozole human menopausal gonadotrophin intrauterine insemination cycles with and without spontaneous LH surge. Reprod BioMed Online (2022) 46(3):566–76. doi: 10.1016/j.rbmo.2022.11.003 36456392

[30] Orvieto R Mohr-Sasson A Blumenfeld S Nahum R Aizer A Haas J. Does a large (>24 mm) follicle yield a competent Oocyte/Embryo? Gynecol Obstet Invest (2020) 85(5):416–9. doi: 10.1159/000510876 32966987

[31] Bezerra FTG Dau AMP Van Den Hurk R Silva JRV. Molecular characteristics of oocytes and somatic cells of follicles at different sizes that influence *in vitro* oocyte maturation and embryo production. Domest Anim Endocrinol (2021) 74:106485. doi: 10.1016/j.domaniend.2020.106485 32858464

[32] Ciepiela P Dulęba AJ Kario A Chełstowski K Branecka-Woźniak D Kurzawa R. Oocyte matched follicular fluid anti-mullerian hormone is an excellent predictor of live birth after fresh single embryo transfer. Hum Reprod (2019) 34(11):2244–53. doi: 10.1093/humrep/dez186 31725884

[33] Carosso AR van Eekelen R Revelli A Canosa S Mercaldo N Benedetto C. Women in advanced reproductive age: Are the follicular output rate, the follicle-oocyte index and the ovarian sensitivity index predictors of live birth in an IVF cycle? J Clin Med (2022) 11(3):859. doi: 10.3390/jcm11030859 35160310 PMC8836866

[34] Alviggi C Conforti A Esteves SC Vallone R Venturella R Staiano S. Understanding ovarian hypo-response to exogenous gonadotropin in ovarian stimulation and its new proposed marker-the follicle-To-Oocyte (FOI) index. Front Endocrinol (Lausanne) (2018) 9:589. doi: 10.3389/fendo.2018.00589 30386293 PMC6199413

[35] Cohen Y Tannus S Alzawawi N Son WY Dahan M Buckett W. Poor ovarian response as a predictor for live birth in older women undergoing IVF. Reprod BioMed Online (2018) 36(4):435–41. doi: 10.1016/j.rbmo.2018.01.008 29478839

[36] Fàbregues F Ferreri J Méndez M Calafell JM Otero J Farré R. *In vitro* follicular activation and stem cell therapy as a novel treatment strategies in diminished ovarian reserve and primary ovarian insufficiency. Front Endocrinol (Lausanne) (2020) 11:617704. doi: 10.3389/fendo.2020.617704 33716954 PMC7943854

[37] Soto N Iñiguez G López P Larenas G Mujica V Rey RA. Anti-mullerian hormone and inhibin b levels as markers of premature ovarian aging and transition to menopause in type 1 diabetes mellitus. Hum Reprod (2009) 24(11):2838–44. doi: 10.1093/humrep/dep276 19643804

[38] La Marca A Broekmans FJ Volpe A Fauser BC Macklon NS. Anti-mullerian hormone (AMH): what do we still need to know? Hum Reprod (2009) 24(9):2264–75. doi: 10.1093/humrep/dep210 19520713

[39] Ubaldi FM Cimadomo D Vaiarelli A Fabozzi G Venturella R Maggiulli R. Advanced maternal age in IVF: Still a challenge? the present and the future of its treatment. Front Endocrinol (Lausanne) (2019) 10:94. doi: 10.3389/fendo.2019.00094 30842755 PMC6391863

[40] Capalbo A Hoffmann ER Cimadomo D Ubaldi FM Rienzi L. Human female meiosis revised: new insights into the mechanisms of chromosome segregation and aneuploidies from advanced genomics and time-lapse imaging. Hum Reprod Update (2017) 23(6):706–22. doi: 10.1093/humupd/dmx026 28961822

[41] Chen X Lin D Ye Y Zhang X Chen D. Trends in the prevalence, prenatal diagnosis, and outcomes of births with chromosomal abnormalities: a hospital-based study in zhejiang province, china during 2014-2020. Orphanet J Rare Dis (2022) 17(1):446. doi: 10.1186/s13023-022-02594-1 36550515 PMC9783762

[42] Mikwar M MacFarlane AJ Marchetti F. Mechanisms of oocyte aneuploidy associated with advanced maternal age. Mutat Res Rev Mutat Res (2020) 785:108320. doi: 10.1016/j.mrrev.2020.108320 32800274

